# ME2 Promotes Proneural–Mesenchymal Transition and Lipogenesis in Glioblastoma

**DOI:** 10.3389/fonc.2021.715593

**Published:** 2021-07-23

**Authors:** Mengting Yang, Xi Chen, Junyao Zhang, Ermeng Xiong, Qianqian Wang, Wenjing Fang, Li Li, Fei Fei, Aihua Gong

**Affiliations:** ^1^ Department of Cell Biology, School of Medicine, Jiangsu University, Zhenjiang, China; ^2^ Immune Regulation and Cancer, Max Delbrück Center for Molecular Medicine in the Helmholtz Association, Berlin, Germany

**Keywords:** malic enzyme 2, glioblastoma, proneural–mesenchymal transition, lipogenesis, AMPK

## Abstract

Malic enzyme 2 (ME2) catalyzes the formation of pyruvate from malic acid and is abnormally expressed in some tumors. However, the exact effects of ME2 on proneural–mesenchymal transition (PMT) and lipogenesis in glioblastoma multiforme (GBM) remain unexplored. Here, we found that ME2 expression was significantly higher in GBM than in normal brain tissues and negatively correlated with overall survival of patients with GBM. Furthermore, we demonstrated that ME2 was positively correlated with mesenchymal features in GBM and promoted proliferation, migration, and invasion of glioma cells. Moreover, ME2 upregulated the expression of mesenchymal markers (N-cadherin, vimentin, YKL40, and MET), whereas it inhibited the expression of proneural maker OLIG2, indicating that ME2 might promote PMT in GBM. We also found that ME2 inhibited the production of mitochondrial reactive oxygen species and AMPK phosphorylation, resulting in SREBP-1 maturation and nuclear localization and enhancing the ACSS2 lipogenesis pathway. Taken together, these results suggest that ME2 promotes PMT and is linked with reprogramming of lipogenesis *via* AMPK–SREBP-1–ACSS2 signaling in GBM. Therefore, ME2 has potential as a new classification marker in GBM and could provide a new approach to glioma treatment.

## Introduction

Glioblastoma multiforme (GBM) is the most common and fatal malignant primary brain tumor in adults, with a 5-year survival rate of less than 5% (1). Central nervous system tumors are classified as grade I to IV by the World Health Organization (WHO) according to histological and molecular characteristics, and the most advanced (grade IV) astrocytomas are classified as GBM (2–4). Moreover, the different GBM phenotypes, which include proneural (PN), neural, classical, and mesenchymal (MES) phenotypes, are relevant to the clinical treatment of recurrent brain tumors (5–7). The MES phenotype is the most malignant and is associated with the worst prognosis, and the PN phenotype tends to transform into the MES phenotype (8, 9). This PN–MES transition (PMT), a process similar to the epithelial–mesenchymal transition (EMT) that occurs in a variety of cancers, is a key event driving invasion, tumorigenesis, and development of gliomas (10–12). In PMT, MES markers (including MET, vimentin, N-cadherin, and YKL-40) are positively correlated with tumor grade growth, metastasis, and progression, whereas PN markers (E-cadherin, OLIG2) are not (13). Despite these findings, the precise mechanism of PMT remains undetermined.

Multiple metabolic pathways create a complex network that promotes the cross-communication between oncogenic and tumor metabolic pathways and promotes tumor progression (14). AMP-activated protein kinase (AMPK) acts as an energy sensor in regulating cellular energy and plays a key role in the upregulation of catabolism and inactivation of anabolism (15). Accumulating evidence has highlighted that lipid metabolism reprogramming is a hallmark of cancer, contributing to cancer transformation and glioma progression (16–19). Sterol-regulatory element binding protein 1 (SREBP-1), an intracellular cholesterol sensor located in the endoplasmic reticulum, positively regulates lipogenesis (20, 21). Cytoplasmic acetyl-CoA is the central precursor of lipid biosynthesis, produced by ATP-citrate lyase (ACLY) from mitochondria or acetyl-CoA synthetase short-chain family member 2 (ACSS2). The ACSS2 reaction can compensate for the loss of ACLY (22, 23). Previous studies have confirmed that malic enzyme 2 (ME2), a key enzyme in the tricarboxylic acid (TCA) cycle, is abnormally expressed in some malignant tumors, including melanoma (24), non–small-cell lung cancer (25), and pancreatic cancer (26). Moreover, ME2 is involved in a series of processes, including tumor growth, metastasis, and malignant transformation (27–29). In addition, ME2 has a profound effect on lipogenesis and glutamine metabolism; however, its role in the PMT process in GBM has not previously been investigated (28).

In this study, we demonstrated that the mechanism of ME2 promoted PMT and lipogenesis. We found that ME2 is associated with overall survival of patients and promotes the proliferation, migration, and invasion of glioma cells. Importantly, to our knowledge, ME2 promoted PMT of glioma cells and reprogrammed lipogenesis *via* the AMPK–SREBP-1–ACSS2 pathway. These results suggest that ME2 is a promising candidate for use in the diagnosis and treatment of GBM.

## Materials and Methods

### Clinical Database Analysis

Gene expression and clinical data of 542 patients with glioma were obtained from The Cancer Genome Atlas (TCGA), Oncomine (https://www.oncomine.org), and the Chinese Glioma Genome Atlas (http://www.cgga.org.cn). We applied the data to create heatmaps, and we added conditional formatting to Excel cells to show colored heatmaps. We analyzed the correlation between ME2 and various indicators and sorted them by category. Then, we sorted the data according to the clinical molecular classification of glioma and classified by color to obtain the heatmap with Excel (Microsoft, Redmond, CA) drawing software.

### Cell Culture

Human glioma cell lines SW1783, U251MG, LN229, and U87MG were obtained from ATCC (Manassas, VA). Cells were cultured at 37°C in a 5% CO_2_ atmosphere and were maintained in high-glucose Dulbecco’s modified Eagle’s medium (DMEM; HyClone, Beijing, China) containing 10% fetal bovine serum (Gibco, Carlsbad, CA). All of the cell lines were authenticated by short-tandem-repeat profiling.

### Quantitative Real-Time Polymerase Chain Reaction (qRT-PCR)

RNAiso Plus (Invitrogen, Carlsbad, CA) was used to isolate total RNA from tumor cells. Reverse transcription and RT-PCR were performed using a RevertAid First Strand cDNA Synthesis Kit (Vazyme, Nanjing, China) and a SYBR GREEN PCR Kit (Vazyme, Nanjing, China) according to the manufacturers’ specifications. Actin was used as the endogenous control for qRT-PCR. The qRT-PCR primer sequences were as follows: β-actin forward: 5′-CACCATTGGCAATGAGCGGTTC-3′; β-actin reverse: 5′-AGGTCTTTGCGGATGTCCACGT-3′; ME2 forward: 5′-GCTCAGAACACCTATGGGGA-3′; and ME2 reverse: 5′-CTATTCTGTTATCACAGG-3′. The results were calculated using the 2^-ΔΔCT^ method (30).

### Plasmid Construction

The sh-EGFP, sh-ME2, Flag-Vector, and Flag-ME2 plasmids were previously constructed in our laboratory. The p3×FLAG-Myc-CMV™14 expression vector and pLKO.1-puro or pLKO-Tet-ON-puro vector were purchased from Sigma-Aldrich (San Francisco, CA).

### Cell Transfection

SW1783 and U251MG cells were inoculated in six-well plates at a density of approximately 60% 12 h before transfection, and 2 μg of Flag-Vector or Flag-ME2 plasmid and 5 μL of lipofectamine TM 2000 reagent (Invitrogen, Carlsbad, CA), were added to each well. After 48 h, cells were harvested for use in subsequent experiments.

The psPAx2 and pMD2.G plasmids were co-transfected with sh-EGFP or sh-ME2 into HEK293T cells using lipofectamine TM 2000. The supernatants were collected 48 h and 72 h after transfection. U87MG and LN229 cells were infected with 1 × 10^6^ recombinant lentivirus transduction units in the presence of 8 mg/mL polybrene (Sigma-Aldrich), and cells were selected by puromycin (2 µg/mL) until all cells became nonviable in the blank group.

### Western Blot Assay

Total protein of cells was extracted using a 2× sodium dodecyl sulfate (SDS) loading buffer and was separated by 10% SDS polyacrylamide gel electrophoresis. Then, protein samples were transferred onto polyvinylidene fluoride membranes and blocked with a blocking solution. The membranes were incubated with primary antibodies overnight at 4°C and with secondary antibodies for 1 h, and then membranes were washed with 1× tris-buffered saline with Tween 20 three times. Protein bands were visualized using chemiluminescence (Meilunbio, Dalian, China) and analyzed using ImageJ software. The primary antibodies included ME2 (Abcam, ab139686, 1:1000), MET (CST, 24294, 1:1000), E-cadherin (CST, 3195, 1:1000), N-cadherin (CST, 4061, 1:1000), vimentin (CST, 5741, 1:1000), YKL-40 (BioWorld, BS6564, 1:800), OLIG2 (Santa Cruz, sc-293163, 1:800), SREBP-1 (Abcam, 28481, 1:1000), ATP–citrate lyase (ACLY; CST, 13390, 1:1000), ACSS2 (CST, D19C6, 1:1000), AMPK (CST, 2532, 1:1000), phospho-AMPK (CST, 2531, 1:1000), and β-tubulin (MA5-11732, 1:2000; Thermo Fisher Scientific) antibodies.

### Cell Counting Kit-8 (CCK8) Assay

A total of 2000 transfected cells were placed in 96-well plates and incubated at 37°C for 72 h. CCK8 solution (Vazyme, Nanjing, China) was added (10 μL) to each well, and cells were incubated at 37°C for 2 h. The absorbance of each well at 450 nm was measured.

### Colony Formation Assay

One thousand transfected glioma cells were placed in six-well plates. Cells were cultured for 15 days in a routine environment, and the medium was changed every 3 days. The cells were observed every day until visible cell colonies appeared. Colonies were fixed with 4% paraformaldehyde for 30 min, stained with crystal violet for 30 min, and then washed with phosphate-buffered saline (PBS; HyClone, Beijing, China). The numbers of colonies were counted under a microscope (MoticAE2000).

### Transwell Migration and Invasion Assays

For the migration assay, 8 × 10^4^ transfected cells were resuspended in serum-free medium and placed in the upper chamber. For the invasion assay, BD Matrigel basement membrane (BD Bioscience, Corning, NY) was added to the upper chamber; the other steps were consistent with the migration assay. The chambers were incubated for 14 h in culture medium with 10% fetal bovine serum in the bottom chambers. Then, the upper chamber was fixed with 4% paraformaldehyde at 4°C for 30 min and stained with crystal violet for 30 min. Finally, the cells were imaged under an inverted microscope.

### Wound Healing Assay

Transfected cells were cultured overnight in a 24-well plate at a density of 1 × 10^5^ cells per well. When the degree of cell fusion was approximately 90%, a scrape wound was created across the diameter with a 10-μL pipette tip. The medium was changed to serum-free DMEM, and the selected location was photographed. Photographs were taken at the same position 24 h later to calculate the relative distance of cell movement. The experiment was repeated three times, and the mean value was calculated.

### Mitochondrial Citric Acid and Pyruvate Assays

Transfected cells were placed in a 24-well plate at a density of 1 × 10^4^ cells per well. The medium was replaced with serum-free medium after 24 h, and cells were incubated at 37°C for 0, 2, 4, and 6 h. Citric acid and pyruvate levels were measured with a citric acid and pyruvate assay kit (Jiancheng, Nanjing, China) according to the manufacturer’s instructions.

### Tissue Microarray Immunohistochemistry

Immunohistochemistry was used to detect the protein expression of ME2 in a human glioma tissue microarray. The tissue microarray was purchased from Easy Biotrade (Nanjing, China). The human glioma samples used in this study were confirmed by a pathologist according to the WHO criteria. The use of human specimens was approved by the relevant institutional review boards.

Tissue sections were air-dried, deparaffinized, and rehydrated. Endogenous peroxidase activity was blocked for 10 min with H_2_O_2_ (3%). Antigen retrieval was performed with citrate buffer in a pressure cooker for 10 min. A 5% serum-free protein block from Biosharp (Nanjing, China) was used to block nonspecific antibody binding for 20 min. Then, slides were incubated overnight at 4°C with primary antibody and at room temperature for 30 min with a species-specific secondary antibody (SA1020; BOSTER, Wuhan, China). Finally, DAB (AR1022; BOSTER, Wuhan, China) was used to develop the color for 10 min, after which distilled water was added to stop the color development. Similar tissue sections immune-stained with nonspecific IgG were used as negative controls. Protein expression was quantified using a four-value intensity score (0, none; 1, weak; 2, moderate; and 3, strong) and a four-value percentage score (0, <10%; 1,10%–40%; 2,40%–70%; 3, >70%).

### Immunofluorescence

Glioma cells were uniformly inoculated in a 24-well plate at a density of 5 × 10^4^ cells per well. After 16–18 h of culture, MitoProbe™ JC-1 (Thermo Fisher Scientific, MA,CA) working solution was added to the wells at a concentration of 300 μL/well, followed by incubation in the dark at 37°C for 10 min. Then, the culture solution was removed by suction and the cells were washed three times with preheated PBS. The cells were added to 500 μL of a preheated PBS solution and immediately observed under a fluorescence microscope for imaging. ImageJ software was used to analyze the fluorescence intensity in the fluorescence images under the same parameter settings, and the relative fluorescence intensity of the experimental group compared with the control group was calculated.

### Statistical Analysis

All data are presented as the mean ± standard error of the mean (SEM). Comparisons between groups were analyzed using the Student’s t test (two groups) or the one-way analysis of variance (ANOVA; multiple groups) using Prism 8.0 software (GraphPad, San Diego, CA). P<0.05 represented a statistically significant difference. All experiments were repeated at least three times.

## Results

### ME2 Is Highly Expressed in GBM and Is Associated With Overall Survival

**Figure 1:**
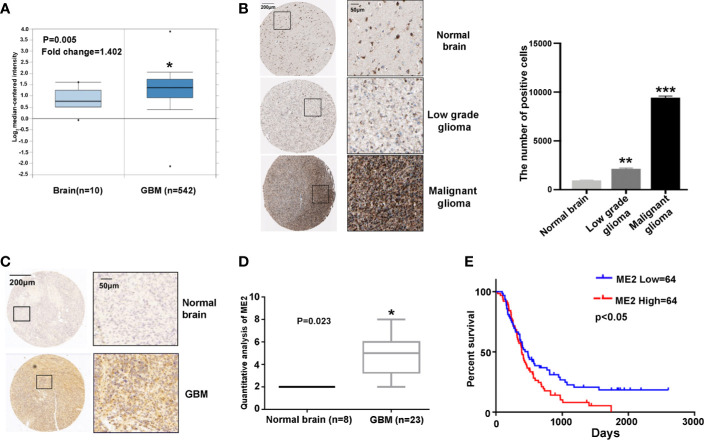
ME2 expression is upregulated in GBM and associated with overall survival of patients. **(A)** mRNA levels of ME2 in GBM and normal brain tissues (*P < 0.05). **(B)** ME2 expression levels in different grades of glioma and statistics and analysis of positive cells results (**P < 0.01, ***P < 0.0001). **(C)** Immunohistochemical staining of ME2 in GBM and normal brain tissues. **(D)** Protein expression score statistics and analysis of staining immunohistochemical results (*P < 0.05). **(E)** Association of ME2 expression with overall survival of patients with GBM (cutoff for ME2 low/high=20.17, P = 0.0423).

Using TCGA data, normal brain tissue samples (n=10) and GBM tissue samples (n=542) were analyzed. The results confirmed that ME2 mRNA expression was higher in GBM tissue than in normal brain tissue (**Figure 1A**). In addition, ME2 expression gradually increased from normal brain tissue to low-grade glioma and then to malignant glioma (**Figure 1B**). The tissue microarray results and the score analysis of staining results also indicated that ME2 expression in normal brain tissue was lower than that in GBM. (**Figures 1C, D** **)**. According to CGGA data, Kaplan–Meier survival analysis showed that patients with GBM and high ME2 expression had shorter survival times (**Figure 1E**). These results suggest that ME2 is highly expressed in GBM and that its expression is negatively correlated with the total survival time of patients with GBM.

### ME2 Is Positively Correlated With MES Features in GBM

**Figure 2:**
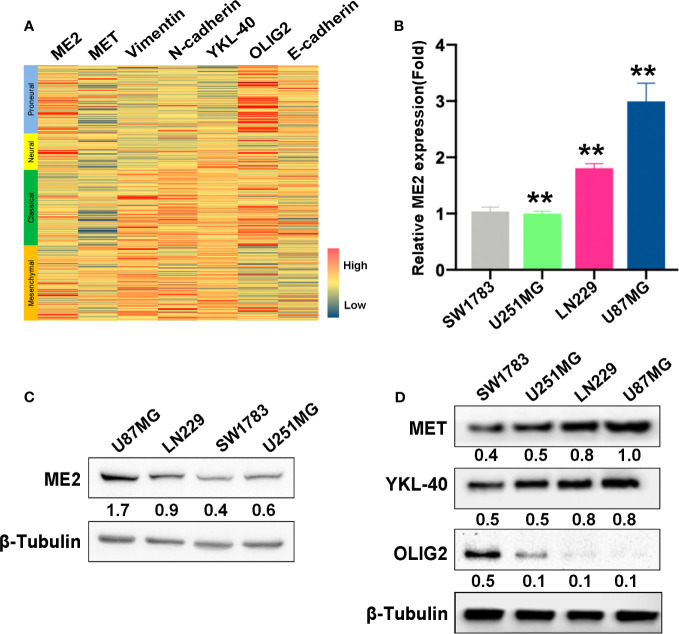
ME2 is positively correlated with mesenchymal features in GBM. **(A)** Gene expression heat map showing the expression of ME2 and PN and MES markers in different gliomas. **(B)** mRNA expression levels of ME2 detected by real-time PCR in SW1783, U251MG, LN229, and U87MG cells (n = 4, **P < 0.01). **(C)** Protein expression levels of ME2 in SW1783, U251MG, LN229, and U87MG cells detected by western blotting. **(D)** Expression of MES markers MET and YKL-40 and PN marker OLIG2 detected by western blotting in SW1783, U251MG, LN229, and U87MG cells.

To investigate the association of ME2 expression with GBM subtypes, a gene expression heatmap was used to display the expression of ME2, MES markers, and PN markers. This investigation revealed that ME2 expression was positively associated with MES markers (including MET, vimentin, N-cadherin, and YKL-40) and negatively associated with PN markers (OLIG2, E-cadherin) (**Figure 2A** and **Table S1**). To confirm these findings, RT-PCR and western blot assays were used to detect ME2 expression in different glioma cell lines. The results indicated that ME2 had higher expression in LN229 and U87MG cells compared with SW1783 and U251MG cells (**Figures 2B, C** **)**. The relative protein expression of MES markers MET and YKL-40 and the PN marker OLIG2 was detected in four different human glioma cell lines. The expression levels of MET and YKL-40 in LN229 and U87MG cells were higher than those in SW1783 and U251MG cells, whereas OLIG2 had lower expression in LN229 and U87MG cells; these results suggest that SW1783 and U251MG cells are associated with the PN phenotype, whereas U87MG and LN229 cells are associated with the MES phenotype (**Figure 2D**). Therefore, ME2 is highly expressed in MES-phenotype glioma cells and is positively correlated with MES features in GBM.

### ME2 Promotes the Proliferation of Glioma Cells

**Figure 3:**
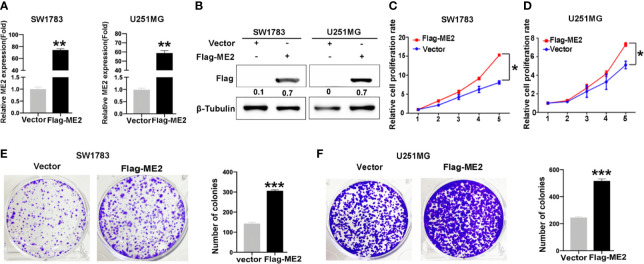
Overexpression of ME2 promotes proliferation of GBM cells. **(A)** ME2 overexpression detected by real-time PCR in SW1783 and U251MG cells (**P < 0.01). **(B)** Western blotting to confirm ME2 expression in in SW1783 and U251MG cells transfected with vector or Flag-ME2 plasmids. **(C, D)** Effects of ME2 overexpression on proliferation of SW1783 and U251MG cells determined by CCK-8 assay (*P < 0.05). **(E, F)** Colony formation assay to determine the colony formation of glioma cells (***P < 0.0001).

**Figure 4:**
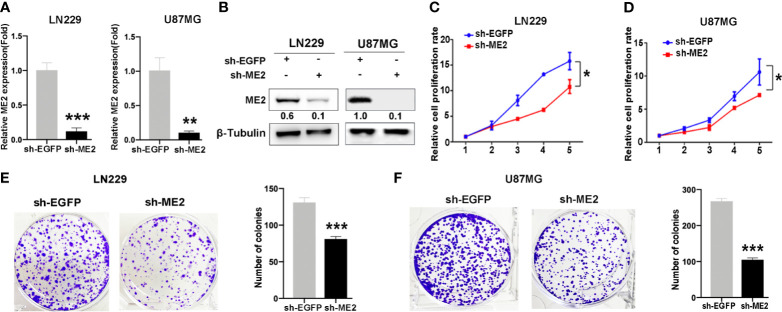
Knockdown of ME2 inhibits cell proliferation in LN229 and U87MG cells. **(A)** Confirmation of the efficiency of ME2 knockdown in LN229 and U87MG cells transfected with sh-EGFP or sh-ME2 plasmids by real-time PCR (**P < 0.01, ***P < 0.0001). **(B)** Western blot to confirm ME2 expression in LN229 and U87MG cells transfected with sh-EGFP or sh-ME2 plasmids. **(C, D)** CCK-8 assay to determine the effects of ME2 overexpression on proliferation of LN229 and U87MG cells (*P < 0.05). **(E, F)** Colony formation assay to determine the colony formation of ability in the above glioma cell types (***P < 0.0001).

To determine the biological function of ME2, SW1783 and U251MG cells (PN phenotype) were transfected with a vector or Flag-ME2 plasmids. The mRNA and protein expressions of ME2 in the experimental group were significantly increased compared with those of the control group (**Figure 3A, B** **)**. Then, the effects of ME2 on proliferation of glioma cells were evaluated by CCK8 and colony formation assays. The results showed that overexpression of ME2 resulted in much greater proliferation ability compared with the vector group in SW1783 and U251MG cells (**Figures 3C–F**). Moreover, when ME2 was knocked down in LN229 and U87MG cells, we found that both mRNA and protein expression of ME2 were significantly reduced in the sh-ME2 (knockdown) group compared with the control group (**Figures 4A, B** **)**. CCK8 and colony formation assays were used to confirm proliferation ability; the results suggested that ME2 knockdown suppressed the proliferation of LN229 and U87MG cells (**Figures 4C–F**). Overall, these results demonstrate that ME2 has a major effect on cell proliferation.

### ME2 Promotes the Migration and Invasion of Glioma Cells

**Figure 5:**
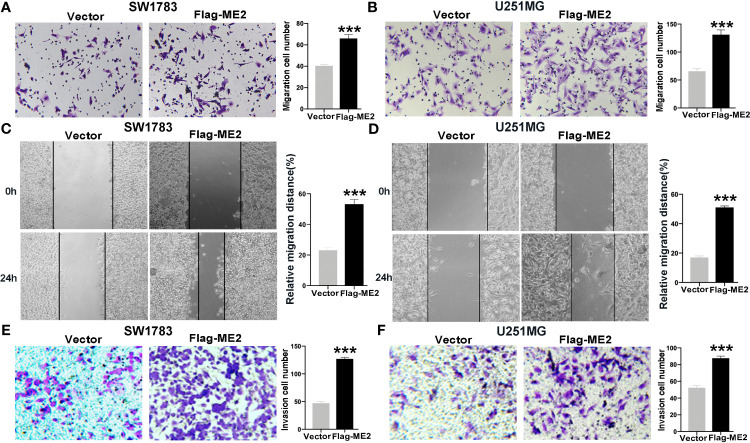
Overexpression of ME2 promotes migration and invasion of GBM cells. **(A)** Representative images of the migration of SW1783 cells transfected with vector or Flag-ME2 plasmids (***P < 0.0001). **(B)** Numbers of migrated cells after Flag-ME2 transfection in U251MG cells (***P < 0.0001). **(C, D)** Wound healing assays to evaluate the effects of ME2 on migratory ability of SW1783 **(C)** and U251MG **(D)** cells (***P < 0.0001). **(E, F)** Invasion ability of SW1783 **(E)** and U251MG **(F)** cells transfected with vector or Flag-ME2 plasmids determined by transwell invasion assay (***P < 0.0001).

**Figure 6:**
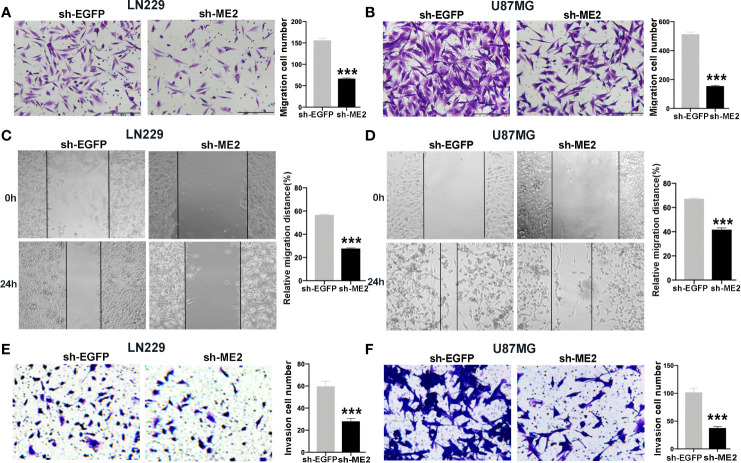
Knockdown of ME2 inhibits migration and invasion of GBM cells. **(A)** Representative images of the migration of LN229 cells transfected with vector or Flag-ME2 plasmids (***P < 0.0001). **(B)** Numbers of migrated cells after Flag-ME2 transfection in U87MG cells (***P < 0.0001). **(C, D)** Representative images of the migration distances of LN229 and U87MG cells transfected with sh-EGFP or sh-ME2 plasmids (***P < 0.0001). **(E, F)** Invasion ability of LN229 and U87MG cells transfected with sh-EGFP or sh-ME2 plasmids determined by transwell invasion assay (***P < 0.0001).

To evaluate the effects of ME2 on cell migration ability, transwell chamber and wound healing assays were conducted. The migration ability in the ME2 overexpression group was significantly stronger than that in the control group (**Figures 5A–D**), and the number of invading cells was higher in the Flag-ME2 group compared with the control group, indicating that ME2 promoted cell invasion in SW1783 and U251MG cells (**Figures 5E, F** **)**. Overall, upregulation of ME2 significantly promoted proliferation, migration, and invasion of the PN-phenotype SW1783 and U251MG cells. We also knocked down ME2 in LN229 and U87MG cells and found that this suppressed migration (**Figures 6A, D** **)**. Furthermore, we found that knockdown of ME2 inhibited invasion ability using a transwell BD Matrigel invasion assay (**Figures 6E, F** **)**. These results confirm that ME2 promotes proliferation, migration, and invasion of GBM cells.

### ME2 Promotes PMT of Glioma Cells

**Figure 7:**
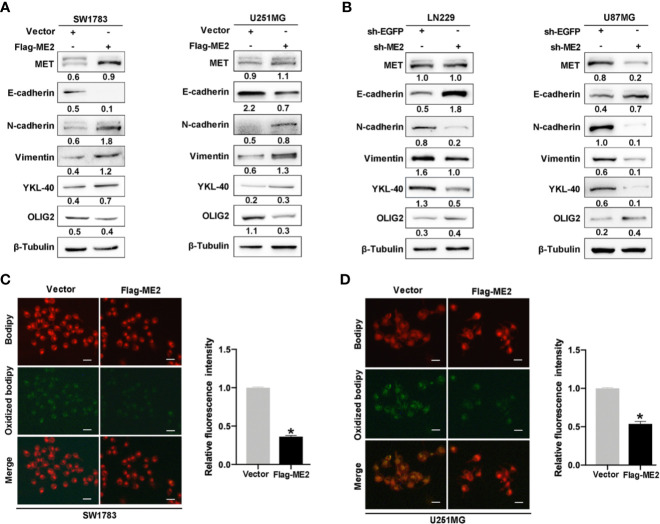
ME2 promotes PMT and inhibits the production of ROS in mitochondria. **(A, B)** Western blots confirming the expression of MET, E-cadherin, N-cadherin, vimentin, YKL-40, and OLIG2 **(A)** in SW1783 and U251MG cells transfected with vector or Flag-ME2 plasmids and **(B)** in LN229 and U87MG cells transfected with sh-EGFP or sh-ME2 plasmids. **(C, D)** Changes in ROS in mitochondria of SW1783 and U251MG cells transfected with vector or Flag-ME2 plasmids detected by fluorescent probe. Green fluorescence represents lipid components oxidized by ROS; red fluorescence represents nonoxidized lipid components. Fluorescence intensity statistics and analysis by Image J (*P < 0.05).

It has been suggested that PMT occurs during relapse in patients with PN-phenotype tumors; this process is extremely detrimental to clinical treatment (31). To determine whether ME2 promotes PMT of glioma cells, we performed a western blot assay to detect the expression of MES markers MET, YKL40, N-cadherin, and vimentin; epithelial marker E-cadherin; and PN marker OLIG2. The results showed that ME2 overexpression in SW1783 and U251MG cells upregulated the expression of N-cadherin, vimentin, YKL40, and MET expression, whereas it downregulated the expression of E-cadherin and OLIG2, suggesting that ME2 promotes PMT of glioma cells (**Figure 7A**). As expected, ME2 knockdown had the opposite effects in U87MG and LN229 cells (**Figure 7B**). Because ME2 is essential for reactive oxygen species (ROS) homeostasis, we examined the effects of ME2 on ROS oxidation in glioma cells using a MitoProbe™ JC-1 fluorescent probe. Green fluorescence intensity was decreased in the Flag-ME2 group compared with the control group, suggesting that ME2 inhibited the production of ROS in mitochondria (**Figures 7C, D** **)**. Thus, ME2 promotes PMT of glioma cells and inhibits the production of mitochondrial ROS.

### ME2 Promotes Lipogenesis *via* the AMPK–SREBP-1–ACSS2 Pathway

**Figure 8:**
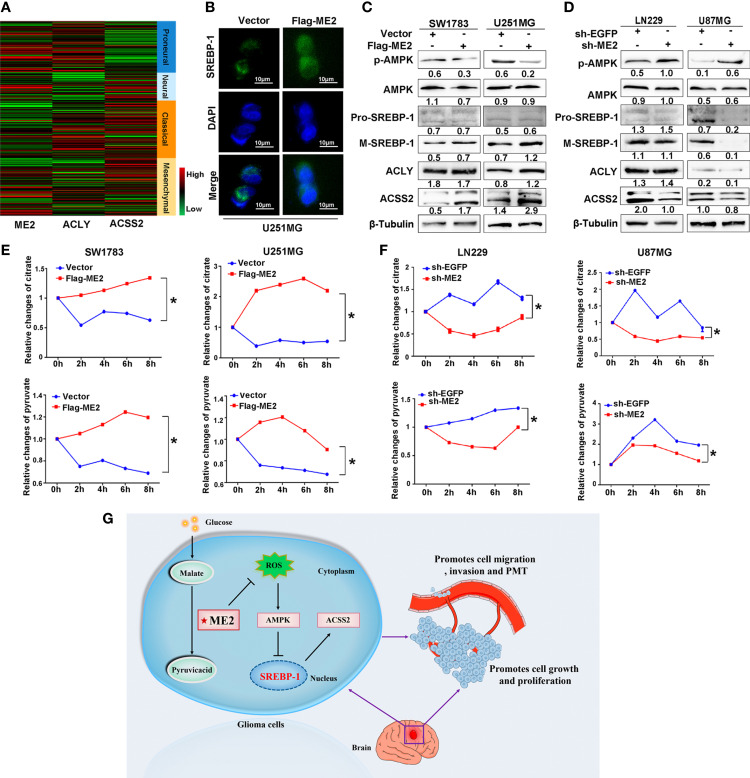
ME2 promotes lipogenesis reprogramming in glioma cells. **(A)** Gene expression heat maps showing expression of ME2, ACLY, and ACSS2 in different gliomas. **(B)** Localization of SREBP-1 in U251MG cells observed by immunofluorescence staining. **(C, D)** Relative changes in citrate and pyruvate concentration **(C)** in SW1783 and U251MG cells and in **(D)** LN229 and U87MG cells transfected with various plasmids (*P < 0.05). **(E, F)** Changes in expression of proteins including p-AMPK, AMPK, pro-SREBP-1, M-SREBP-1, ACLY, and ACSS2 in **(E)** SW1783 and U251MG cells and in **(F)** U87MG and LN229 cells transfected with various plasmids. **(G)** Mechanistic diagram of the role of ME2 in GBM.

We also investigated the effects of ME2 on the lipogenesis pathway in glioma cells. Existing studies have confirmed that acetyl-CoA is the material for lipid synthesis and that acetyl-CoA develops from the ACLY pathway and the ACSS2 pathway (32). The heatmap revealed that ACLY was highly expressed in the PN phenotype, whereas ACSS2 was highly expressed in the MES phenotype of GBM (**Figure 8A** and **Table S2**). The precursor of SREBP-1 (pro-SREBP-1) migrates to Golgi and releases the N-terminal domain with transcriptional activity through a series of proteolytic processes. The mature SREBP-1 (M-SREBP-1), an active nuclear form of SREBP, is then translocated into the nucleus, which activates the transcription of SREBP response genes, promoting the lipogenic process (33). We used immunofluorescence to examine the location of SREBP-1 in cells and found that overexpression of ME2 increased its nuclear localization (**Figures 8B** and **S1**). A western blot assay was then used to detect the effects of ME2 expression on lipid metabolism–related proteins in glioma cells. As a result of proteolytic processes, the molecular weight of pro-SREBP-1 is 120 kDa, whereas the weight of M-SREBP-1 is only 68 kDa. The western blot results showed that ME2 overexpression increased the expression levels of ACSS2 and M-SREBP-1 but decreased those of phosphorylated AMPK (p-AMPK) (**Figure 8C**). Moreover, downregulation of ME2 decreased the expression of ACSS2 and M-SREBP-1, whereas it increased the expression of p-AMPK (**Figure 8D**). No significant changes in ACLY expression were observed during these experiments.

The mitochondrial malic enzyme ME2 catalyzes malate to pyruvate and then promotes the citrate cycle that is linked to fatty acid synthesis (34). We determined the changes in mitochondrial citric acid and pyruvate concentrations by chemiluminescence in glioma cells. The concentrations of mitochondrial pyruvate and citric acid were higher in the Flag-ME2 group than in the vector group, indicating that ME2 promoted the production of pyruvate and citric acid (**Figure 8E**). ME2 knockdown had the opposite effect (**Figure 8F**). Therefore, we suggest that ME2 enhances *de novo* synthesis of fatty acids mainly *via* the AMPK–SREBP-1–ACSS2 pathway in glioma (**Figure 8G**).

## Discussion

In this study, we confirmed that ME2 promotes cell proliferation, migration, and invasion, consistent with previous findings (35). Importantly, we found that ME2 was correlated with the GBM MES phenotype and promoted PMT and reprograming of the lipogenesis pathway *via* AMPK–SREBP-1–ACSS2 in glioma cells. These results suggest that ME2 is a candidate for use in the molecular diagnosis of GBM.

PMT in GBM is an EMT-like process that usually occurs in patients after radiation therapy and chemotherapy (36–38). Evidence suggests that PMT is driven by certain restricted master regulators, including STAT3, C/EBPβ, and TAZ (39–42). Moreover, FoxM1 can promote acquisition of MES features and PMT in glioma *via* activation of the EGFR–AKT–GSK3β signaling pathway (43). Recently, pyruvate kinase 2, a key enzyme in glucose metabolism, was shown to have significantly higher expression in the MES subtype of GBM compared with the PN subtype, suggesting that the metabolic pathway of GBM may be reprogrammed when PMT occurs (44–46). Previous studies have indicated that ME2 promotes growth, transformation, and metastasis of malignant tumors (24–26). Here, we found that ME2, a metabolic enzyme in the TCA cycle, is critical for PMT of GBM and is linked to lipogenesis (47).

Uncontrolled regulation of cellular energy and metabolic reprogramming are important characteristics of malignancy including EMT and PMT (48–50). Studies have shown that GBM metabolic phenotypes not only undergo glycolytic pathway reprogramming but also undergo changes in the mitochondrial TCA cycle and lipogenesis pathways (22). The *de novo* synthesis of fatty acids has emerged as a source of therapeutic targets for cancers (51). The main substrate for fatty acid synthesis is cytoplasmic acetyl-CoA, which can be derived from either ACLY-mediated citrate conversion or ACSS2-mediated acetate conversion (52, 53). ACLY serves as a cross-link between the TCA cycle and the *de novo* synthesis of fatty acids by catalyzing the formation of acetyl-CoA (54). In this study, we found that ME2 could increase the production of mitochondrial pyruvate and citric acid but had little effect on the expression of ACLY. This implies that ACLY has no effect on the *de novo* synthesis of fatty acids. Furthermore, ME2, a metabolic enzyme involved in the TCA cycle, significantly affected the expression of ACSS2, which converts acetate to acetyl-CoA independently of the TCA cycle. Previous studies have confirmed that ME2 is essential for ROS homeostasis and energy change (26, 55). Therefore, it is reasonable to conclude that ME2 may influence ROS production and AMPK phosphorylation. AMPK is a highly conserved metabolic regulator that can maintain the intracellular energy balance under physiological metabolic stress conditions (56). As expected, we found that ME2 inhibited the production of ROS and AMPK phosphorylation. Recent studies have indicated that activated AMPK could inhibit the maturation and nuclear translocation of SREBP-1 and the subsequent transcriptional activation of ACSS2 (57, 58). We confirmed that ME2 promotes SREBP-1 maturation and nuclear localization and increases ACSS2 expression, indicating that ME2 upregulates *de novo* lipogenesis, probably *via* AMPK–SREBP-1–ACSS2, in glioma cells.

In conclusion, ME2 promotes PMT of GBM cells, and ACSS2 has an important role in the ME2-induced *de novo* synthesis of fatty acids in glioma cells. These results suggest that ME2 could provide a new approach for glioma diagnosis and treatment.

## Data Availability Statement

The datasets presented in this study can be found in online repositories. The names of the repository/repositories and accession number(s) can be found in the article/**Supplementary Material**.

## Author Contributions

MY, XC, and AG conceived the idea and designed the study. MY was responsible for writing the manuscript. QW drew the hypothesis diagram. All other listed authors participated in the experiments and data collection. All authors contributed to the article and approved the submitted version.

## Funding

This study was supported by the National Natural Science Foundation of China (81772694, 81372718).

## Conflict of Interest

The authors declare that the research was conducted in the absence of any commercial or financial relationships that could be construed as a potential conflict of interest.

## Publisher’s Note

All claims expressed in this article are solely those of the authors and do not necessarily represent those of their affiliated organizations, or those of the publisher, the editors and the reviewers. Any product that may be evaluated in this article, or claim that may be made by its manufacturer, is not guaranteed or endorsed by the publisher.

## Supplementary Material

Supplementary Figure 1 Quantification of the average SREBP-1 nuclear/cytoplasmic fluorescence ratio per cell in **Figure 8B** (*P < 0.05).
