# Supplementary figures and images for: ME2 Promotes Proneural–Mesenchymal Transition and Lipogenesis in Glioblastoma
Source: Front Oncol. 2021 Jul 23;11:715593. doi: 10.3389/fonc.2021.715593 (PMC8351415; doi:10.3389/fonc.2021.715593)

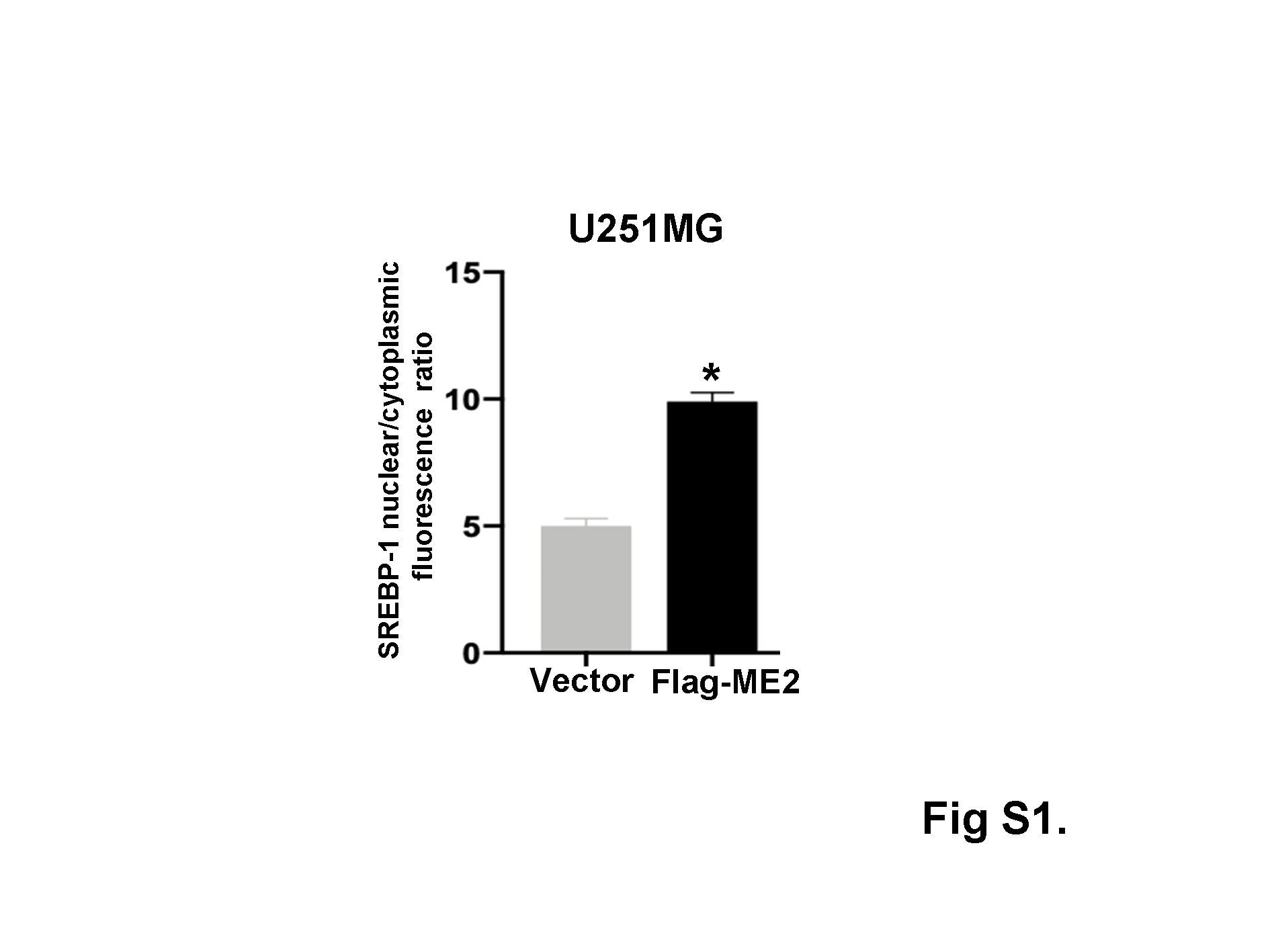

Supplement: Supplementary Figure 1 — Quantification of the average SREBP-1 nuclear/cytoplasmic fluorescence ratio per cell in Figure 8B (*P < 0.05). [file Image_1.tif]
